# Seasonal variation in food availability and relative importance of dietary items in the Gambian epauletted fruit bat (*Epomophorus gambianus*)

**DOI:** 10.1002/ece3.5150

**Published:** 2019-04-15

**Authors:** Kofi Amponsah‐Mensah, Andrew A. Cunningham, James L. N. Wood, Yaa Ntiamoa‐Baidu

**Affiliations:** ^1^ Centre for African Wetlands University of Ghana Accra Ghana; ^2^ Institute of Zoology Zoological Society of London London UK; ^3^ Disease Dynamics Unit University of Cambridge Cambridge UK; ^4^ Department of Animal Biology and Conservation Science University of Ghana Accra Ghana

**Keywords:** dietary composition, ecosystem function, feeding ecology, fruit bat

## Abstract

1. The Gambian epauletted fruit bat (*Epomophorus gambianus*) is very common across a variety of West African habitats, but very little information is available on its feeding ecology or its contribution to ecosystem function.
2. We investigated seasonal variation in food availability and the relative importance of dietary items used by this species in a forest‐savannah transitional ecosystem. Dietary items were identified from 1,470 samples of fecal and ejecta pellets which had been collected under day roosts or from captured bats over a 2‐year period (2014–2015).
3. Plant phenology studies illustrated strong seasonal correlations between fruiting and flowering and rainfall patterns: Fruits were available throughout the year but with peaks of abundance during the rainy season, while flowers were mostly abundant during the dry season. *Epomophorus gambianus* bats utilized fruit and flower resources from 30 plant species. Although the plant species used depended on seasonal availability, there were clear preferences for certain species.
4. Flowers were an important food source for this fruit bat species especially during the dry season, contributing up to 79% of dietary items when fruit abundance was low. Ficus fruits were also important food item for *E. gambianus*, constituting over 40% of all dietary samples identified.
5. *Policy implications*. Our results show the importance of flowers in the diet of *E. gambianus* and highlight this species as an important pollinator and seed disperser, including for economically and ecologically important plant species. These findings contribute to an improved understanding of the ecological importance and potential role of this species in the forest‐savannah transition ecosystem for the development of fruit bat conservation management strategies.

## 1 INTRODUCTION

Dietary studies can increase our understanding of the interrelationships between animals and their environment and how individual species affect and contribute to their ecosystems (Stier & Mildenstein, 2005). Fruit bats (Pteropodidae) are almost exclusively phytophagous, relying extensively on fruits, flowers, and leaves (Aziz, Olival, Bumrungsri, Richards, & Racey, 2016; Marshall, 1985). Through their feeding interactions with plants, many fruit bat species provide vital ecosystem services and are, therefore, regarded as keystone species. The biology of bats, particularly their reproduction and migration, is influenced by the distribution and timing of food availability (Marshall, 1985), which varies across landscapes and seasons (Cumming & Bernard, 1997). The timing of fruiting/flowering is often irregular across landscapes, which makes it difficult to generalize findings (such as reproductive synchrony with food abundance) obtained from one landscape to others (Cumming & Bernard, 1997). The utilization of food resources is also not uniform among fruit bats, and some studies, for example Baker and Harris (1957), Marshall and McWilliam (1982) and Barclay and Jacobs (2011), have shown inter‐ and intraspecific variations in food utilization in some African members of the Pteropodidae.

Very few studies have explored the relative use and importance of different food items in the diet of fruit bats (Marshall, 1985; Stier & Mildenstein, 2005). Most studies that explored the dietary resources of fruit bats failed to quantify the relative use of identified dietary resources, primarily providing lists of food items and apparently assuming equal use (Stier & Mildenstein, 2005). The current lack of knowledge on the relative use of food items limits efforts to assign trophic roles to fruit bats (Marshall, 1985), resulting in inadequate characterization of the importance of their roles in the functioning of ecosystems. This limits our understanding of how bats impact ecosystems at local or larger scales and how changing land use and habitat modifications affect bat populations (Stier & Mildenstein, 2005; Wood et al., 2012).

**Figure 1:**
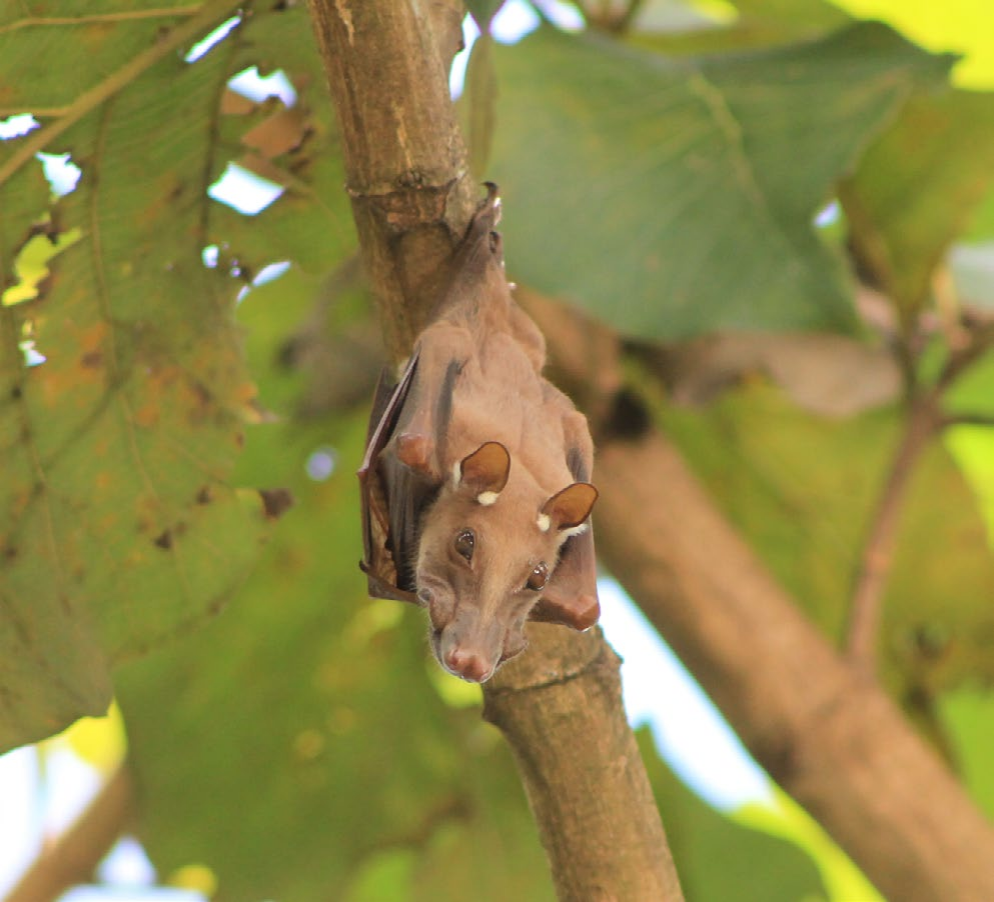
A Gambian epauletted fruit bat (*Epomophorus gambianus*). Credit: Kofi Amponsah‐Mensah

*Epomophorus gambianus* (family Pteropodidae) is a medium sized fruit bat that is widespread throughout much of West Africa (Figure 1). The species is commonly described as a generalist and opportunistic fruit feeder that thrives well in degraded forests and a variety of human modified habitats. We studied the diet of this widespread species in a West African forest‐savannah transition ecological zone, and here, we describe the seasonal availability and relative use of food items for this species. Our findings contribute to an improved understanding of the ecological importance and potential role of this species in the forest‐savannah transition ecosystem for the development of fruit bat conservation management strategies.

## 2 MATERIALS AND METHODS

### 2.1 Study area

Data for this study were collected in Golokuati, a town located in the Volta region of Ghana (N 06°59.851′ E 000°26.218′) from January 2014 to December 2015. A large *E. gambianus* colony (ca. 5,000 bats) that roosts in trees within the town was the focus of this study. The vegetation of the area is a transition between semi‐deciduous forest and Guinea savannah woodland, but it has undergone significant changes mainly due to agricultural use, timber exploitation, and human settlement, resulting in a heterogeneous landscape with remnant pockets of the original forest, regenerating secondary forest and farm bush. The area experiences the wet semi‐equatorial climate where rainfall occurs in a double maxima pattern (annual range 1,016–1,210 mm),but this is changing gradually to a single extended rainy season (late April–October) and a 4–5 months dry season between November and April (Ghana Statistical Service, 2014). The mean annual temperature is 29°C, ranging between 26 and 32°C.

### 2.2 Diet identification by fecal and ejecta sample analysis

The dietary components of *E. gambianus* were identified from collections of fecal and ejecta pellets under roosting and feeding trees and from captured bats. Fecal samples were collected by placing 1.5 × 1 m plastic sheets directly under day roosts of *E. gambianus* during the early hours of the day (0500–0900 hr). To avoid repeated collection of fecal samples from the same individuals that could arise from splatters or discontinuous deposition, feces that were within 5 cm and had same characteristics (color, texture) of already collected material were ignored (Stier & Mildenstein, 2005). Fresh ejecta pellets were collected under feeding roosts within the study area. Fecal/ejecta sample collection was done on two to three consecutive days in each sampling month for 21 months.

Bats captured through mist‐netting for other studies also provided opportunities to collect additional samples and data on dietary sources for *E. gambianus*; trapping was done between 1900 and 0500 hr using ground mist nets. Any whole or remnants of fruits or feces or ejecta pellets expelled by *E. gambianus* bats during the process were collected opportunistically for the dietary study. Bats trapped were checked also for the presence of fresh pollen on their nostrils or wings, or for remnants of flower parts in their mouths. The occurrence of pollen/flower parts on the body or in the mouth of a bat was used as a proxy for feeding on flower resources by that bat and was counted as a “flower resource” sample.

We conducted informal interviews with local farmers to help locate plants known to be utilized by bats in West Africa (Marshall, 1985) and also to identify plants which they knew to be utilized by bats. Plants identified by the local farmers were monitored for visits by *E. gambianus* to confirm their use as a food source. Other fruiting and flowering trees in the study area were also monitored for visits and feeding activity by *E. gambianus* bats.

Each ejecta and fecal sample was collected separately into a clean, new plastic bag using a wooden spatula. Latex gloves were worn during fecal and ejecta pellet collection process. Each sample collected was washed through a 0.3 mm sieve using tap water in the field before examining for seeds, flower parts, and other food particles with the aid of a handheld magnifying glass. A reference collection based on fruits and seeds, and their characteristics (color, smell, texture) of fruiting plants occurring in the study area was developed and used to identify the fruits and seeds collected from the fecal and ejecta samples (Djossa, Fahr, Kalko, & Sinsin, 2008; Picot, Jenkins, Ramilijaona, Racey, & Carriere, 2007; Stier & Mildenstein, 2005). Seeds from up to five different species of fig that occurred at the study site were very similar and could not be identified to the species level. Hence, all fig seeds from ejecta/fecal samples were pooled together as “*Ficus* spp.” Each dietary material identified in each fecal or ejecta sample was recorded to occur just once in that sample (i.e., as presence only without quantification within a sample) as it was impossible to quantify the number or quantity of particular food items within each fecal/ejecta sample collected. Both fecal and ejecta samples were treated as equal and dietary items identified from both were treated the same and pooled together. Dietary materials identified from samples were expressed as relative abundance (percentage) of total monthly samples collected, with the assumption that each sample collected was from an individual bat. All dietary materials were assumed to be equal; the calorimetric or energetic content of dietary items was not determined in this study.

### 2.3 Estimation of monthly food resource availability and abundance, and the timing of food resource abundance in relation to rainfall

To estimate availability and seasonal variations in food resources, plants identified as being utilized by *E. gambianus* were monitored monthly for fruiting and flowering abundance. Fruit/flower abundance was determined through visual estimation (Chapman et al., 1992) by a single observer (KAM) throughout the study to maintain consistency and to increase internal validity. Fruit/flower abundance was estimated by a modification of the method described by Devineau (1999), which entailed categorization of flowering and fruiting phases into four stages (0–3) with corresponding phenology scores of 0, 0.09, 0.5, and 1 (Table 1). For each plant monitored, the total number of fruits and flowers on the plant was estimated each month. Monthly fruit/flower abundance per plant was then estimated as: (Total number of fruits/flowers present on the plant) × (Phenology score) for each month. To account for unequal numbers of each plant species that was monitored, monthly fruit/flower abundance was expressed as an average of the total number of plants monitored for each plant species.

**Table 1:** Categorization and description of flowering and fruiting stages of plants and their corresponding phenology scores used in quantifying fruit and flower abundance

Fruiting/flowering stage	Stage description	Estimated % of fruits/flowers matured	Phenology score
0	No flower/fruit present; or, if present, all flowers and fruit dead and desiccated	0	0
1	Flower buds with less than 10% of flowers open; early fruit setting with less than 10% of fruits matured	9	0.09
2	Flower buds present and 10% to 50% of flowers opened; 10% to 50% of fruits matured size	50	0.5
3	Peak flower bloom with over 50% of flowers opened peak of fruit maturity with over 50% of fruits fully matured	100	1

We tested for an association between food availability and dietary use using spearman ranked correlation coefficient (ρ). We used the estimated percentage of fruits/flowers that are matured on each monitored plant (as described in Table 1) as a measure of food abundance rather than the estimated monthly total number of fruits on each plant. We chose this measure of abundance because early fruiting/flowering stages of plants produce high numbers of fruits/flowers which are immature and not readily available for use by bats, therefore, using these estimates will produce false‐negative correlations.

To determine the timing of flower and fruit abundance in relation to rainfall within the study area, we related mean monthly fruit and flower abundance to the mean monthly rainfall for the study area. Rainfall data for the study area were obtained from the Ghana Meteorological Agency (www.meteo.gov.gh).

## 3 RESULTS

### 3.1 Food composition and relative abundance of food resources based on fecal and ejecta sample analysis

A total of 1,470 samples of fecal and ejecta pellets were collected over the 21‐month sampling period, comprising 401 samples collected from trapped bats, 505 ejecta pellets, and 564 fecal samples collected under day roosts. A total of 129 trapped bats were observed with fresh pollen on their bodies/nostrils and 866 fecal and ejecta samples collected contained seeds. Plant material in 42 fecal samples could not be identified and did not match any of the descriptions in the reference collection. These were not included in the analysis.

Thirty species of plant, belonging to at least 16 families, were identified in the *E. gambianus* fecal and ejecta samples examined (Table 2). Six species were utilized for their flowers only, 20 for their fruits, and four for both fruits and flowers. Three to eight different food items were utilized in each month. There was no significant difference in the use of specific dietary items by the different sexes of *E. gambianus* that were trapped. Flower resources, together with fruits from four plant groups, *Vitex doniana*, *Anthocleista vogelii, Ficus* spp., and *Polyalthia longifolia*, constituted over 80% of all dietary items identified*. Ficus* spp. were identified in over 40% of all samples collected and were utilized in all sampling months (Table 3). Fruits of *Solanum* sp. and *Melothria* sp. were recorded in 11 and seven of the sampling months, respectively, but had low frequencies of 2.4% and 1.1% of the total samples collected. *Vitex doniana* occurred most frequently in the diet of *E. gambianus* from August to October when the variety of food sources identified was low relative to the rest of the year. Fruits of *A. vogelii* and *P. longifolia* constituted significant proportions of the dietary items identified during March/April and May/June, respectively. Flower resources were recorded in 10% of all samples collected and were most common from November to February.

**Table 2:** Plant species and their food resource identified as being consumed by *Epomophorus gambianus*

Plant family	Species	Common name	Food resource	Source
Anacardiaceae	*Spondias mombin*	Yellow mombin	FR	a,b,c
	*Anacardium occidentale*	Cashew	FR	b,c
	*Mangifera indica*	Mango	FR	a,b,c
Annonaceae	*Polyalthia longifolia*	Indian mast tree	FR	a,b,c
	*Annona muricata*	Soursop	FR	b,c
Bignoniaceae	*Spathodea campanulata*	African tulip tree	FL	a,b
Bombacaceae	*Bombax buonopozense*	Red silk‐cotton	FL	b
	*Ceiba pentandra*	Silk Cotton	FL	a,b
Caricaceae	*Carica papaya*	Pawpaw	FR	a,b,c
Combretaceae	*Terminalia catappa*	Indian almond	FR	a,b,c
Cucurbitaceae	*Melothria* sp	Mouse melon	FR	a
Fabaceae	*Daniellia oliveri*	African copaiba balsam	FL	a,b
	*Parkia biglobosa*	African locust bean	FL	a,b
Gentianaceae	*Anthocleista vogelii*	Cabbage tree	FR, FL	a,b
Malvaceae	*Adansonia digitata*	Baobab	FL	b
	*Sterculia rhinopetala*	Brown sterculia	FR	a
Meliaceae	*Azadirachta indica*	Neem	FR, FL	a,b,c
Moraceae	*Antiaris toxicaria*	False iroko, Antiaris	FR	a,b
	*Ficus* spp. (five species)	Figs	FR	a,b,c
	*Milicia excelsa*	Iroko, Odum	FR	a,b,c
Musaceae	*Musa* sp.	Banana	FR, FL	a,c
Myrtaceae	*Psidium guajava*	Guava	FR	a,b,c
	*Syzygium* sp	woodland waterberry	FR	a,b
Solanaceae	*Solanum* sp.	Potato tree	FR	a,b
Verbenaceae	*Vitex doniana*	Black plum	FR	a,b,c
*		Unidentified	FR, FL	a

Note

FL: flower; FR: fruit; source refers the origin(s) of information presented: a: current study; b: literature Marshall (1985); Marshall and McWilliam (1982); c: indigenous knowledge;

* Unidentified trees.

**Table 3:** Monthly relative use (%) of dietary items identified from *Epomophorus gambianus* fecal and ejecta pellets over a 21‐month period. No data were collected in September and October 2014 and January 2015

	Month/frequency of occurrence (%)																				
	Jan	Feb	Mar	Apr	May	Jun	Jul	Aug	Nov	Dec	Feb	Mar	Apr	May	Jun	Jul	Aug	Sep	Oct	Nov	Dec
Dietary Item	2014										2015										
*Anthocleista vogelii*			49	64	15	1					2.9	17	1.6	18	24	3.2					
*Antiaris toxicaria*											5.7										
*Azadirachta indica*							23	1.9	1.6			1.3	3.6	16		3.2	1.9				
*Carica papaya*									6.3	2.8											
*Ficus* spp.	31	2.7	26	32	8.3	4	29	24	45	51	6	64	75	6.3	18	52	35	12	39	47	22
Flower resources	41	79						5.8	33	4.3	26									13	62
Insect parts						3.3															
*Mangifera indica*					2.8	3						14	4.3	3							
*Melothria* sp.	6.9						1.5		3.1	1.4					6.7			7.6		6.7	
*Milicia excelsa*			1.1	4.5								2.6									
*Musa* sp.								3.8													
*Polyalthia longifolia*					69		27						6.3	25	47	6.5					
*Psidium guajava*			12					19			2.9	2				16	4.7				
*Solanum* sp.					4.2		1.5		4.7	4.2	2.9					3.2	6.6	3.5	4.8	2	14
*Spondias mombin*							7.6														
*Sterculia rhinopetala*														1.3							
*Vitex doniana*							3.3	46	6.3							6.5	47	78	52	13	1.6
others	2.7		12			17	7.6							3.8	4.4	9.7	4.7		4.8		
Number of dietary resources	4	2	5	3	5	5	8	6	7	5	6	6	5	7	5	8	6	4	4	5	4

**Figure 2:**
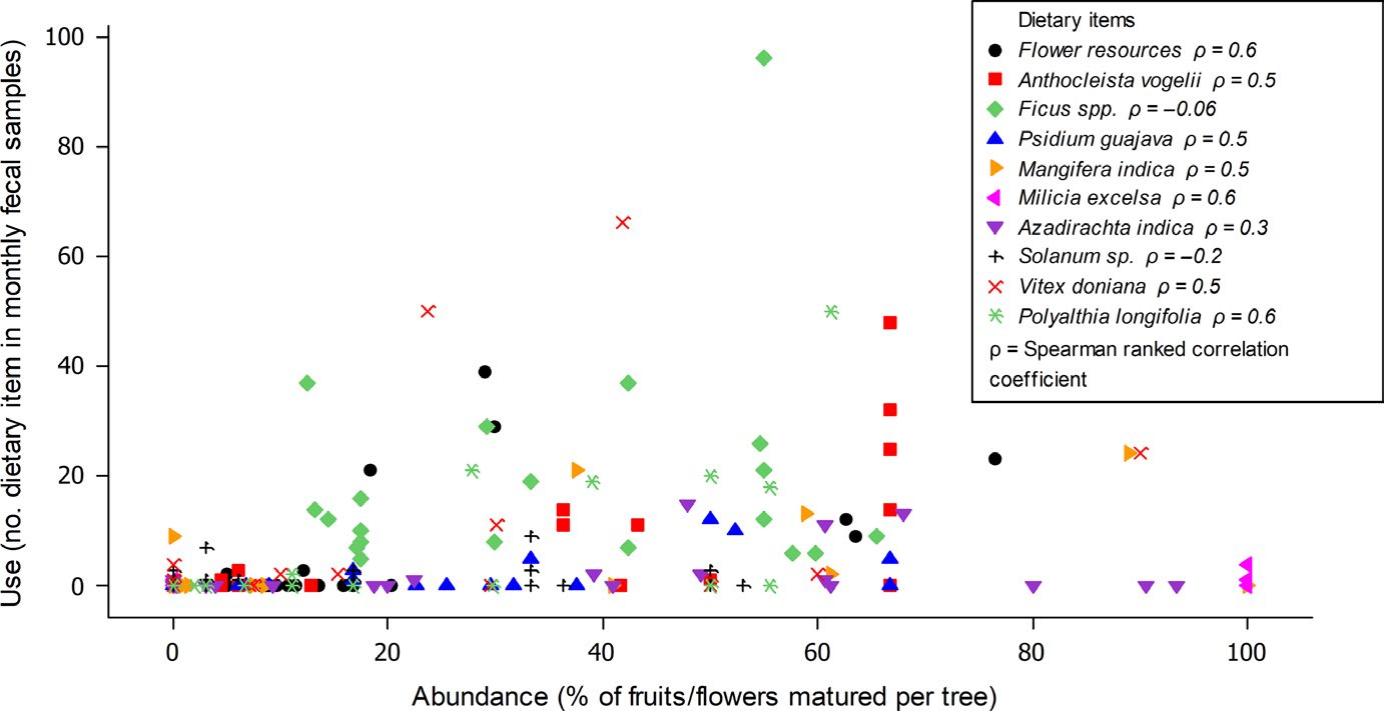
Correlations between the abundance and use of dietary items of *Epomophorus gambianus*. Spearman ranked correlation coefficient (ρ) values are indicated beside species/food items. Abundance is expressed as the percentage of the estimated fruits/flowers that are matured per tree. The use of dietary items is expressed as the number of times that dietary item is recorded in total monthly fecal collections. Only dietary items that were detected in the diet of the bats in three or more sampling months were used. One outlier value for *Ficus* spp. (use = 288, abundance = 37.5) not shown on graph

The use of most dietary items correlated strongly and positively with their abundance (Figure 2) with fruits and flowers being used when they were available and abundant. The use of both *Ficus* spp. and *Solanum* sp. however was weakly and negatively correlated to their abundance. The use of *Ficus* spp. was mostly relatively higher than that of other food items with similar monthly abundances.

### 3.2 Timing of food resource abundance in relation to rainfall

**Figure 3:**
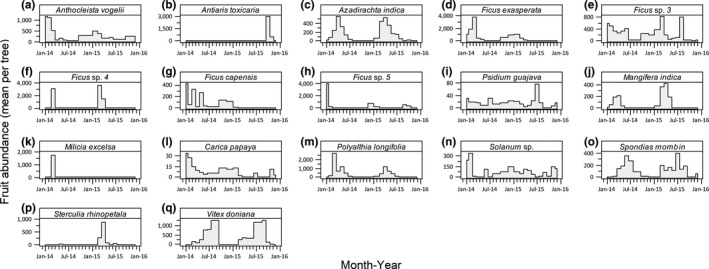
Estimated mean monthly fruiting abundance of plant species whose fruits were identified to be consumed by *Epomophorus gambianus*. Abundance is estimated as the total number of fruits × monthly phenology score per tree

Twenty‐two plant species were monitored for the timing and abundance of fruits and/or flowers. Fruits of many species, for example, *Psidium guajava*, *Solanum* sp, *A. vogelii,* and *Ficus* spp., were available throughout the sampling period but with varying fruit abundances (Figure 3). The fruits of *Sterculia rhinopetala*, *Antiaris toxicaria*, and *Milicia excelsa* were not consistently available throughout the year.

**Figure 4:**
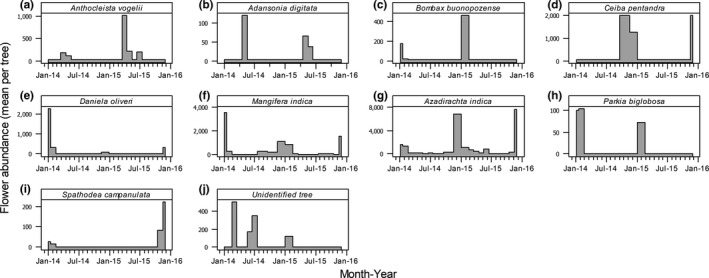
Estimated mean monthly flowering abundance of plants whose flowers were identified to be utilized by *Epomophorus gambianus*. Abundance is estimated as total number of flowers × monthly phenology score per tree

Flowering in most of the plants exploited by *E. gambianus* occurred once per year around December/January. A few species, for example, *Mangifera indica* and *Azadirachta indica*, had two flowering periods; a major one in December/January and a minor one in June/July (Figure 4). Flowers lasted for short periods and most flowering plants were devoid of flowers within a month of flowering.

**Figure 5:**
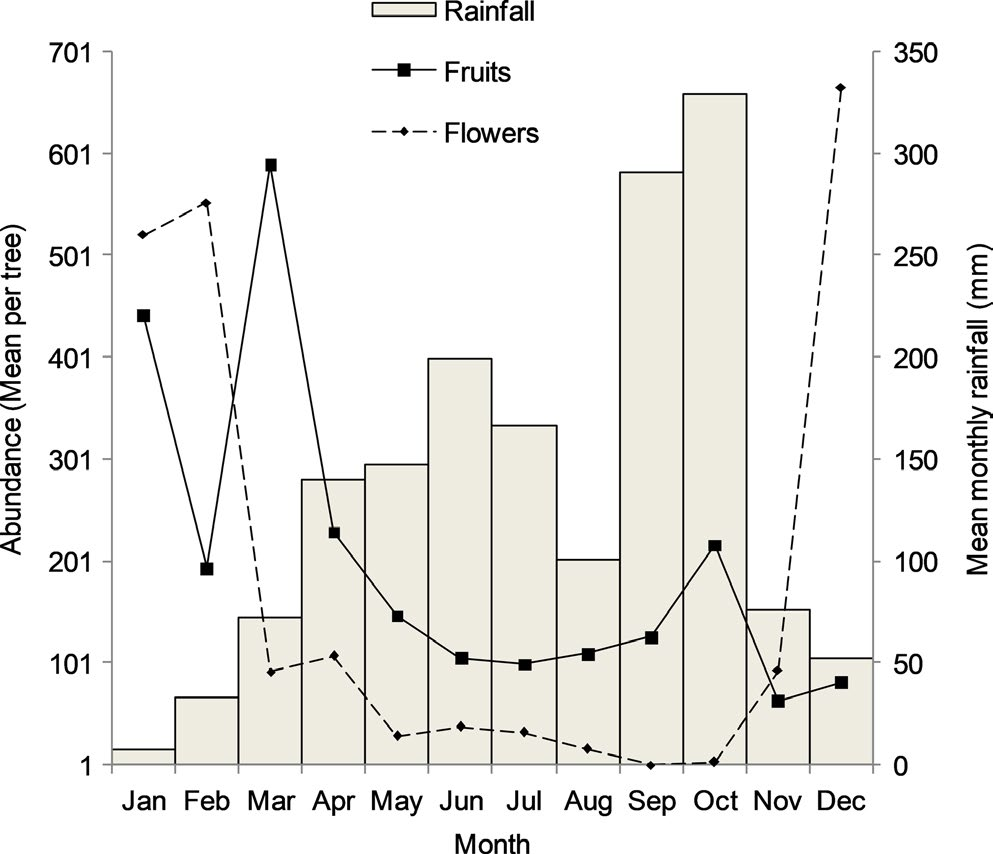
Abundance and the timing of flowering and fruiting in relation to mean monthly rainfall. Combined mean monthly fruit and flower abundance over 2 years were used

Mean monthly rainfall in the study area was high over a continuous 7‐ to 8‐month period with peaks in June and October. These two peaks in rainfall were separated by a short period of reduced rain in August and a 5‐month‐long dry season following the October peak (Figure 5). The main period of flower abundance occurred during the dry season, November to February, with a peak in December. A lower incidence of flowering occurred from April through to August with minimum flower abundance around September–October. Compared to flowers, fruits were relatively more available throughout the year, with a major peak in fruit abundance occurring in March and a minor peak in October.

## 4 DISCUSSION

### 4.1 Dietary composition

The diet of *E. gambianus* within our study area in Ghana, West Africa, was found to consist of 30 plant species from at least 16 families. These results confirm the varied diet of this species (Boulay & Robbins, 1989; Marshall, 1985; Marshall & McWilliam, 1982). We were unable to identify only 42 samples from 1,470 that did not match our reference collection, possibly because they were eaten outside our sampling area; therefore, our results provide a conservative estimate of the dietary sources available to *E. gambianus* in this forest‐savannah transition zone.

With the exception of the family Cucurbitaceae, all of the plant families we identified were already known to be eaten by fruit bats in West Africa (Marshall, 1985; Marshall & McWilliam, 1982; Mickleburgh, Hutson, & Racey, 1992; Rosevear, 1965). Fruits of *Melothria* sp. (family Cucurbitaceae) were identified in about 1% of the total samples and in samples from seven months of the study. Although there is no record of this plant family as a food source of fruit bats in West Africa, Marshall (1985) lists the fruits and leaves of *Sechium* sp., another member of the family Cucurbitaceae, as a food source for the bat genus *Cynopterus* in parts of Asia. Our observation of *Melothria* sp. in the diet of *E. gambianus* provides an addition to the extensive range of plant species and families recorded as being utilized by fruit bats in West Africa.

Most food items identified as part of the diet for *E. gambianus* appeared generally to be linked to the seasonal availability of these food items within the study area, thus supporting the description of this bat species as an opportunistic feeder (Boulay & Robbins, 1989; Marshall, 1985; Marshall & McWilliam, 1982). Although seasonal availability may dictate the use of food resources in fruit bats, some species can also exhibit preferential diet selection. For example, Baker and Harris (1957) described *Eidolon helvum* as preferentially eating the flowers of *Ceiba pentandra* over those of *Parkia clappertoniana*, when both were in flower. In the current study, fruits of *Solanum* sp and *Melothria* sp were identified in the diet of *E. gambianus* during several months, but their overall relative abundance was less than 4% of samples. Fruits of *Ficus* spp. had a relatively high frequency of occurrence in dietary samples of up to 52% in June–July when fig fruits were at the lowest abundance and as many as nine other food items were available. Thus, fruits of *Ficus* spp. may be preferred over other food items and searched for during the period of low fig fruit abundance. This implies the preference for some dietary items over others or use in hierarchical manner, where some food items may only be supplementary to more preferred ones. This would support Marshall's suggestion that *E. gambianus* and most other fruit bats could be spatio‐temporal “sequential specialists,” with a preference for some food items among those available, rather than true generalists (Marshall, 1985).

### 4.2 Relative importance of food of different plant items in the diet of *Epomophorus gambianus*

*Ficus* spp. were represented in over a third of all samples and were eaten in all months of this study, indicating that fruits from *Ficus* spp. constitute an important component of the *E. gambianus* diet. The genus *Ficus* is an important source of food in the diet of other frugivorous animals, including over 20 genera of fruit bat (Mickleburgh et al., 1992), so it is not surprising that it featured in the diet of *E. gambianus* in the current study. *Ficus* fruits might be an important source of food because multiple species occur with an asynchronous fruiting phenology, resulting in fruits being widely available throughout the year (Barclay & Jacobs, 2011; Shanahan, So, Compton, & Corlett, 2001). Our observations are consistent with this; *Ficus* spp. fruits were available throughout the study period. *Ficus* fruits contain high levels of calcium relative to other fruits (O'Brien et al., 1998), so their year‐round availability could be important for the maintenance of the bats' health, including for pregnant and lactating females (Barclay & Jacobs, 2011).

Other fruits that may play vital roles in the diet of *E. gambianus* include those of *V. doniana* and *A. vogelii*. These fruits accounted for significant proportions of the diet of *E. gambianus,* which suggests preferential selection of these fruits over other food items when available. Fruits of *V. doniana* have a higher protein content (72.8–82.4 g/kg) (Agbede & Ibitoye, 2007; Vunchi et al., 2011), compared to *P. guajava* and *Ficus* spp. (22.2 and 24.6 g/kg, respectively; Ruby, Nathan, Balasingh, & Kunz, 2000) and, thus, *V*. *doniana* fruits may be preferred over other available fruits. The availability and use of the fruits of *V. doniana* and *A. vogelii* coincides with the periods of late pregnancy (March and September) and parturition (April/May and October/November) reported for *E. gambianus* in West Africa (Thomas & Marshall, 1984). Thus, these two plant species together with *Ficus* spp. could be important dietary items during such vital stages in the reproduction of *E. gambianus*.

While fruit bats usually feed mostly on fruits, flowers also constitute important dietary items (Marshall, 1985). However, very few studies have assessed the extent of flowers in the diet of fruit bats. In our study, flower resources contributed over 9% of the dietary items identified. In the dry (“lean” fruiting) seasons, flowers contributed up to 79% of dietary items, although our analyses did not allow us to estimate the proportion of calorific intake in different months We identified the flowers of at least 10 plant species as being utilized by *E. gambianus*, all of which have been reported previously as food sources for other fruit bat species in West Africa (Baker & Harris, 1957; Marshall, 1985; Marshall & McWilliam, 1982; Mickleburgh et al., 1992)

Because flowering and fruiting periods for most of the monitored plants were asynchronous, we assumed that bats observed with fresh pollen on their nostrils and/or wings had visited these plants purposely to feed on nectar and/or pollen from their flowers. Some trapped bats had both pollen and fruit items collected from them (*n* = 18 bats), but in majority of these cases (*n* = 17) the identified fruits were from plants which were not flowering at that time, hence supporting our assumption. Peaks in flower abundance and the use of flower resources occurred mostly during the dry season when fruit abundance was lowest. This suggests that the use of flowers plays an important subsistence rather than a supplementary role in the diet of *E. gambianus*. The flowers of *C. pentandra* for instance are reported to be an important food source for bats in Madagascar during the dry season (Andriafidison et al., 2006), where flowers from a single tree could sustain a large chiropteran community over a short period (Gribel, Gibbs, & Queiroz, 1999). Flowers can contribute considerable amounts of dietary protein (Law, 1992a; Long & Racey, 2007; Nelson, Miller, Heske, & Fahey, 2000; Ruby et al., 2000), supplementing the low protein content of most fruits (Barclay & Jacobs, 2011; Marshall, 1985; Ruby et al., 2000; Stier & Mildenstein, 2005).

Although *E. gambianus* was observed to visit flowering trees and several trapped bats were covered in pollen, we could not confirm if these bats were actively eating pollen, nectar, or both. The importance of pollen in the diet of *E. gambianus* is unclear. Boulay and Robbins (1989) stated that there was no evidence to support *E. gambianus* feeding on pollen, based on the absence of pollen in analyzed gut contents (Baker and Harris (1957). Happold (2013) also suggests that this species visits flowers for their nectar but not the pollen. However, Pteropodidae bats may lick pollen directly from anthers during feeding (Marshall, 1985) or ingest it during grooming of fur dusted with pollen after visiting plants to feed on nectar (Law, 1992b). *Pteropus* spp. may actually feed and depend on pollen as an important food source (Long & Racey, 2007; Marshall, 1985; Mickleburgh et al., 1992). Andriafidison et al. (2006) report that in Madagascar, over 40 percent of the 118 plant taxa that have been identified as part of the diet of *Pteropus rufus* and *Eidolon dupreanum* were identified from pollen in feces. They suggested that the contribution of pollen and nectar to the diet of fruit bats may be largely underestimated and the low reporting of pollen in the diet of bats could be due to a surveillance bias or methodological constraints. We were not equipped to carry out microscopic analysis of pollen; therefore, further studies of the pollen content of feces are needed to confirm its importance as a dietary material in fruit bats.

### 4.3 Timing of food resources

Food availability for African fruit bats is constrained by rainfall as fluctuations in fruit abundance are tied to rainfall patterns (Cumming & Bernard, 1997; Happold & Happold, 1990; Rautenbach, Kemp, & Scholtz, 1988). Fruit phenology studies show an abundance of fruits during the rainy season, while flowering occurs in the preceding dry season (Frankie, Baker, & Opler, 1974; Janzen, 1967). Our findings are consistent with this pattern; however, our findings demonstrate that fruits are available throughout the year, but with peaks in fruit abundance occurring at the onset of, or during, the rainy season. Each fruiting period occurred a month or two after the flowering period. In drier areas of Africa, with stronger seasonal variations in rainfall, seasonality in fruiting and flowering may be more pronounced, causing fruits to be less available throughout much of the year (Cumming & Bernard, 1997). In such areas, peaks in flower abundance might play an even more important role in the diet of frugivorous bats during periods of low fruit abundance.

The timing and seasonality of food abundance are particularly important to the timing of reproduction in fruit bats (Cumming & Bernard, 1997; Happold & Happold, 1990), where birthing is timed such that food is available for juveniles after they are weaned (Cumming & Bernard, 1997; Fleming, Hooper, & Wilson, 1972). In West Africa, annual bimodal peaks in parturition for *E. gambianus* occur in April/May and in October/November (Thomas & Marshall, 1984). Lactation lasts about 7–13 weeks (Nowak, 1999; Thomas & Marshall, 1984), implying that the first annual postweaning period for *E. gambianus* occurs around June/July, coinciding with a period of availability of both flowers and fruits. The second postweaning period occurs around December/January, which coincides with the observed major peak in flower abundance. These observations suggest that flowers might have an important role in supporting the maintenance and growth of newly weaned bats.

### 4.4 Importance of *Epomophorus gambianus* to ecosystem services

Seeds from several of the fruits identified as part of diet of *E. gambianus* were small enough to be easily swallowed undamaged during feeding, retained in the gut of the bats and identified in the fecal samples collected. Even larger seeds that are not swallowed can be carried and dropped some distance from the parent plants. Over 58% of fecal samples collected contained seeds of *Ficus* spp., *M. excelsa, A. vogelii, Solanum* sp., *P. guajava,* and *Melothria* sp These seeds identified in the samples collected did not appear different in size, shape, and morphology from those collected from whole fruits as part of the reference collection. By feeding on these fruits, *E. gambianus* plays a vital ecological role in the successful dispersion of seeds of these plants. Fruit bats are reported to be responsible for the dispersion of seeds from at least 156 species of plants (Fujita & Tuttle, 1991). During the dry season, when flowers are relatively more abundant, the increased utilization of flowers by *E. gambianus* during this period likely makes this bat species an important pollinator of forest and fruit trees, such as *C. pentandra*, *D. olivieri, P. biglobosa, A. vogelii,* and *B*.* buonopozense*.

Several food plants utilized by *E. gambianus*, including *M. excelsa, B. buonopozense, Ficus* spp., *S. campanulata*, and* C. pentandra*, are important pioneer species of the forest‐savannah transition ecological zone (Hawthorne & Gyakari, 2006). These plants contribute substantially to ecosystem biomass, and fruits of plants like *Ficus* spp. provide food for birds and mammals (Kunz, Braun de Torrez, Bauer, Lobova, & Fleming, 2011; Shanahan et al., 2001). Other plants, such as *Solanum* sp.*, Ficus capensis*, and *A. vogelii*, are important species during early succession stages of forestation (Swaine & Hall, 1983). By spreading the seeds of these species, therefore, *E. gambianus* likely plays an important role in the maintenance and regeneration of forest vegetation, and the persistence of original forest plant species. This role could be especially important when considering current levels of forest degradation and loss.

In addition to helping to maintain forest ecosystem function, the feeding activities of *E. gambianus* appear to provide several direct and indirect benefits to people. Some of the plants that are likely to be pollinated and dispersed by *E. gambianus* have high economic importance. Tree species, such as *M. excelsa, B. buonopozense, S. rhinopetala,* and *C. pentandra*, have economic importance in Ghana, being exploited for timber and other uses (Hawthorne & Gyakari, 2006). In particular, *M. excelsa* is heavily exploited to the extent that it is now threatened by overexploitation in Ghana (Hawthorne & Gyakari, 2006; Taylor, Kankam, & Wagner, 2000).

Fruits of plants, such as *V. doniana, S. mombin, Syzygium* sp*.,* and *P*. *guajava*, are sold and consumed locally and contribute to the diet and income of people, especially rural dwellers across West Africa. Even though plants like *M. indica*, *P*. *guajava,* and *Anacardium occidentale* are cultivated on large scales and may no longer rely on bats for their dispersal or pollination, fruit bats such as *E. gambianus* remain relevant for the maintenance of the genetic diversity of their wild types (Kunz et al., 2011). The services provided by *E. gambianus* through its foraging highlight the importance of this species (and fruit bats in general) to the ecosystems in which they occur. Evidence of these services, such as those presented in this study, therefore, should be used to inform the public and policymakers to promote the conservation of *E. gambianus* and other fruit bat species.

## CONFLICT OF INTERESTS

The authors declare that there are no conflict of interests.

## AUTHORS’ CONTRIBUTIONS

YNB conceived the ideas; YNB and KAM designed methodology; KAM collected the data, analyzed the data, and led the writing of the manuscript; YNB, AAC, and JLNW obtained the funding; AAC, JLNW, and YNB supervised the research, reviewed and edited the manuscript. All authors contributed critically to the drafts and gave final approval for publication.

## Data Availability

Dryad https://doi.org/10.5061/dryad.8sk41pc.
